# A study of relationship between social support, work values and job search behavior

**DOI:** 10.3389/fpsyg.2022.1021299

**Published:** 2022-11-25

**Authors:** Fang Wang, Jian-Guo Qu

**Affiliations:** Wulingshan K-12 Educational Research Center, College of Education Sciences, Huaihua University, Huaihua, China

**Keywords:** social support, work values, job search behavior, relationship, labor market

## Abstract

The COVID-19 outbreak has put more pressure on the labor market, reducing employment opportunities and increasing graduate unemployment. Therefore, this study was undertaken to explore the relationship between social support, work values and job search behavior. The theoretical model was tested using the data collected from 560 Chinese fresh graduates (M_age_ = 23.45 years; standard deviation = 2.02). The participants completed questionnaires that assessed their social support, work values and job search behavior. Descriptive statistics and structural equation modeling were used for data analysis. The results indicated that social support was positively and directly associated job search behavior and work value mediated the association between social support and job search behavior. These findings will encourage future researchers to investigate the phenomena of job search behavior.

## Introduction

In recent years, academics from throughout the world have started paying attention to the issue of college students’ employment (Chowdhury and Miah, 2019; Clausen and Andersson, 2019; Lewis, 2019). Due to COVID-19’s effects and the several industries that were affected, China has seen frequent layoffs and unemployment, which has raised the pressure on college students to find jobs (Hensher, 2020; Kawohl and Nordt, 2020). Additionally, effective employment of college students is necessary for the harmony and stability of society, and it is also necessary for the success of college student’s growth. Job searching is increasingly seen as a necessary component of the working world as college students look for employment after graduation or to seek professional options. A meta-analysis that identified job search behavior as a key factor in locating work was conducted at the same time as a sharp surge in studies on job search and unemployment (Kanfer et al., 2001). A dynamic self-regulatory process that starts with the identification and commitment to an employment objective is how Kanfer et al. (2001) characterized job search behavior. This aim then initiates search behavior that is intended to achieve the desired goal. It is suggested that achieving or giving up the employment goal will end the job search process and any related job search attempts and activities. Understanding individual differences as determinants of search behaviors and outcomes or strategies targeted at improving job search outcomes has been the main focus of prior research (e.g., Kanfer et al., 2001; Liu et al., 2014; Probst et al., 2018). The macro events that influence job search behavior, however, have received far less attention in a study (Wanberg et al., 2020). College students are in a difficult condition when it comes to their job search because of the unfavorable macro scenario. Researchers are increasingly aware of the urgent need to examine the job search behavior of college students who are just entering the labor market since the job search process is full of complaints, which has gotten more serious in recent years. Social support is defined as activities taken during interpersonal communications that make the recipients feel valued, appreciated, and cared for (Cobb, 1976). Social support is given and received among social relationships with different degrees of resemblance. It may be used to offer guidance, help with issues, disseminate knowledge, address personal issues, and, when necessary, soothe and encourage (Agneessens et al., 2006). According to Mack and Rhineberger-Dunn (2019), social support is a byproduct of interpersonal work connections that can enhance the recipient’s well-being or coping skills. It can also refer to other people’s constructive acts (Deelstra et al., 2003). Initially, it would appear logical that social support would influence job search behavior. The job search might result in emotions of powerlessness, alienation, and loneliness since it is so time-consuming, stressful, and unpredictable (Teye-Kwadjo, 2021). It highlights the significance of social support in creating a stress-free job search journey when perceived social support in such circumstances helps one overcome a planned job search. Social support is referred to as a motivating phenomenon that improves a job seeker’s job search efforts. It motivates one to continue their job search behavior for a longer period (Schwarzer and Knoll, 2007; Paul and Moser, 2009). The excitement of job searchers to participate in a job search activity is influenced by job search behavior (Vansteenkiste et al., 2013; Maier and Seligman, 2016). According to studies, social support helps job seekers adopt an upbeat mindset. According to the theory of planned job search, job searchers face increased social pressure as a result of their lack of social support, which discourages them from exerting full effort. Additionally, it impairs their ability to cope with stressful situations, which can cause mental and emotional distress (Lakey and Cohen, 2000; Van Hooft and Noordzij, 2009). Additionally, other research showed that social support helps job searchers develop their psychological and social resources (Chen and Fellenz, 2020), which are crucial for coping with the stress, anxiety, and trauma connected to the job search as well as the delay in finding a job (Cohen, 2004; Salanova et al., 2006). McKee-Ryan et al. (2005) provided evidence in their meta-analyses to support the idea that jobless teenagers with social support are better equipped to deal with job loss and get more assistance in finding new employment. Similar to this, Holmstrom et al. (2015) have researched to examine the link between job search behavior, social support, and self-efficacy among new-entry job searchers and jobless or underemployed youngsters. According to the results, job seekers’ self-esteem and self-efficacy are enhanced by perceived social support, which increases their motivation to look for a job more actively (Al-Jubari et al., 2021). Societal support from friends, family, and peers is said to influence people’s professional choices. Additionally, it increases self-efficacy and aptitude for locating suitable employment prospects tried to research 6,987 residents of Trent, England (Roberts et al., 1997). The authors looked at how well job searchers received social support. According to the survey, known people did not provide unemployed youth with adequate social support. Numerous additional kinds of research, including (Lakey and Cohen, 2000; Waters and Moore, 2001; Oh et al., 2014; Hulshof et al., 2020; Al-Jubari et al., 2021), have also documented a variety of beneficial impacts of social support on job seekers.

There are many areas of life where values are implemented, including employment, religion, culture, sports, and politics (Sagie and Elizur, 1996). Values are crucial components of a person’s personal, social, and professional life because they influence our decisions, deeds, attitudes, and behaviors (Elizur et al., 1991; Dose, 1997). Work values are the principles that guide a person’s professional life. Like fundamental values, work values are convictions about desirable outcomes, like high pay, or desirable activities, like communicating with others (Ros et al., 1999). Work values specifically outline what professionals look for in a job in general and what duties are crucial to their job satisfaction (Elizur, 1984; Ros et al., 1999). Examining work-related outcomes, such as career choices (Tourna-Germanou and Kapadaidakis, 2006), job satisfaction (Locke, 1976; Knoop, 1991; Brown and Lent, 2008; Koilias et al., 2012; Bouwkamp-Memmer et al., 2013), organizational socialization, and organizational commitment (Dose, 1997), has been seen as crucially dependent on work values (Meyer et al., 1998).

Since work values represent the degree of worth, relevance, and attractiveness of what occurs in the work setting and also influence people’s decisions and affective reactions, the theory of work values is a valuable conceptual tool for examining the main causes of professional happiness (Locke, 1976). Additionally, values may predict job satisfaction more accurately and consistently than hobbies, skills, and personalities (Rounds, 1990). If values are largely constant (Ravlin and Meglino, 1989), it would be crucial to look at how they affect job search behavior as it would be the main way to establish person-organization value congruence. Values may also be viewed as a requirement or desire for specific outcomes or states (England, 1975). According to Katz (1973), job search behavior might be seen as preferences for environments that permit or encourage the expression of specific values or value systems. Furthermore, Vroom (1966) discovered that people search for jobs that align with their professional objectives. Some of these objectives had a strong moral component (e.g., the potential to progress professionally, the chance to assist society), which suggests that people’s job-search behavior was influenced in part by their values at work. The role of social support provided by parents and peers in the formation of young people’s attitudes and beliefs on employment-related information, such as work value. Studies with elementary (Meelissen and Drent, 2008) and secondary students (Shashaani, 1994) revealed a high correlation between parental support and students’ perceptions of the importance of their work. A mediating element that may impact the social support associated with engaging in job search behavior is work value. College students may be guided by elements of social support, such as advice from others, from the job search stage to the action phase of engaging in job search behaviors.

The main aim of the present research is to examine relationship between social support and job search behavior and the mediating role of work value on the relationship between social support and job search behavior among fresh graduates during the COVID-19 pandemic. Although previous studies found associations between these three variables, the mediation model was never thoroughly tested. Since social support is a positive predictor of work value (Shashaani, 1994; Meelissen and Drent, 2008) and job search behavior (Waters and Moore, 2001; Oh et al., 2014; Holmstrom et al., 2015; Chen and Fellenz, 2020; Al-Jubari et al., 2021), while work value is a strong positive predictor of job search behavior (Katz, 1973; England, 1975), it is expected that social support has an indirect positive impact on job search behavior, *via* work value. Holmstrom et al. (2015) found the mediating effect of job-search self-efficacy on the association between received esteem support and job-search behavior among new-entrant job seekers and unemployed, underemployed, and/or displaced workers. Lee et al. (2017) found the mediating effect of organizational commitment on the relationship between social support and job performance among bank expatriates. However, there is a gap in the research since no studies have been done on the relationships between social support, work values, and job search behavior in China. Limited information on graduates’ job searches was found after a review of current research in the area. The purpose of this study was to investigate the relationship between social support, work values, and job search behavior in Chinese graduate students. Results may offer valuable info that may be used to better understand organizational outputs including social support, work values, and fresh graduates’ job-searching behaviors.

## Hypotheses development

As we discussed in the theory development section, past empirical work supports the idea that social support is widely acknowledged as a crucial coping resource in stressful situations, in general, and during the period of a person’s inability to find a job, in particular. The protective effects of social support can help graduate job seekers deal with stressors associated with job search behavior. Fresh Graduate job seekers would require more social support for job search efforts during and after COVID-19 than they did at any time in China’s recent employment history. Therefore, we formed the following hypothesis:

*Hypothesis 1*: social support will be positively associated with job search behavior.

Job search behavior might be seen as preferences for environments that permit or encourage the expression of specific values or value systems. Vroom (1966) suggests that people’s job-search behavior was influenced by their work values. Social support provided by parents and peers affect the formation of work value of fresh graduates. College students may be guided by elements of social support, such as advice from others, from the job search stage to the action phase of engaging in job search behaviors. Therefore, we formed the following hypothesis:

*Hypothesis 2*: Work value will mediate the relationship between social support and job search behavior.

## Materials and methods

### Participants

The research universities were chosen using the convenience sampling methods. The participants of this research consisted of final-year Chinese undergraduates from a public university in Hunan Province, China who were recruited *via* flyers on campus notices and in classrooms seeking participation in November 2021. The advertisement gave a brief introduction and explained the significance of the research project. The participants were acquainted with the purpose of the research and the anonymity of their responses after scanning the QR code to join was guaranteed. After receiving their consent, the link/QR code to the online questionnaire was emailed to them. The participants received a 3 RMB Hong Bao (literally a “red envelope” that implied a monetary reward) that would be provided to them after they completed the online surveys. 560 valid questionnaires were obtained, with an effective rate of 95.46%. In the valid questionnaires, there were 212 male (37.86%) and 348 female (62.14%). The students ranging from 20 to 27 years old, the average age was 23.45 years, and the standard deviation was 2.02. The average value of the variable was used to fill in the missing data for a small number of surveys.

### Measures

#### Social support rating scale

Social support was assessed using the Chinese version (Xiao, 1994) of SSRS (social support rating scale). The SSRS has three categories and 10 items in total. The scale consists of four factors: objective support (example item: “When things go wrong, I frequently receive sympathy and attendance from family, friends, relatives, or neighbors”), subjective support (example item: “I have pleasant relationships with my neighbors”), support utilization (example item: “I frequently seek aid when I am in difficulty”). The items were graded on a four-point Likert scale with scores ranging from 1(strongly disagree) to (strongly agree). The total score for the 10 items was used to calculate the subject’s current overall social support status, and the score for each category was calculated using the scores for the relevant items. The scale’s Cronbach alpha coefficient was 0.79 in the present study.

#### Work values scale

The Chinese version of work values scale (WVS) developed by Wu et al. (1995) was used to measure individuals’ perceptions of the meaning of work. It has 49 items (e.g., “I can achieve my life’s ambition in my work”) on seven dimensions, with seven questions for each sub-scale: self-growth orientation, self-actualization orientation, dignity orientation, social interaction orientation, organizational security and economic orientation, stability and freedom from anxiety orientation, and leisure health and transportation. All the measures were assessed on a 5-point Likert scale with scores ranging from 1 (very unimportant) to 5 (very important). In the current study, it had a Cronbach’s alpha of 0.88 and for the subscales it ranged from 0.76 to 0.85.

#### Job search behavior scale

Job search behavior was assessed using the Chinese version (Wang et al., 2009) of JSBS (job search behavior scale), which was developed by Kanfer et al. (2001). The JSBS has a total of 25 questions (e.g., “I apply for a job in different institution regardless of information about vacancies”). The items were rated on a 5-point Likert scale, with scores ranging from1 (very rarely) to 5 (very often). The questionnaire is divided into five dimensions, namely job search intensity, short-term and long-term job search, independent job search, job search frequency and relationship job search. The scale’s Cronbach’s alpha of 0.78 in the present study.

### Statistical analysis

Descriptive statistics and correlations were calculated using SPSS 22.0. Additionally, PROCESS macro model 6 (Hayes, 2017) was used to test the mediating effects of resilience and academic burnout. The bootstrapping method was used to produce 95% bias corrected confidence intervals (CI) for the magnitude of these effects based on 5,000 resamples of the data. These effects were regarded as statistically significant if the confidence intervals did not contain zero.

## Results

### Common method bias test

The Harman one-way test was used to test for common method bias (Podsakoff et al., 2003). The results showed that a total of 15 factors had characteristic roots greater than one and the variance explained by the first factor was 24.95%, which was less than 40%, indicating that there was no severe common method bias in this study.

### Descriptive statistics and correlation analysis

The correlation coefficients between the variables and the results of descriptive statistical analysis are shown in Table 1. Social support is significantly and positively associated with work values and job search behavior; work values are significantly and positively correlated with job search behavior.

**Table 1:** Descriptive statistics and correlations between variables (*n* = 560).

	*M*	*SD*	1	2	3
1 Social support	33.46	3.63	1		
2 Work values	2.98	0.38	0.64**	1	
3 Job search behavior	3.07	0.83	0.68**	0.57**	1

**p < 0.01.

### Testing the mediating effects

To test the mediation effect, we ran a two-step regression analysis (Wen and Ye, 2014). Social support entered at first step of regression model 1. At second step, social support and work value were entered simultaneously of regression model 2. Results presented in Table 2 indicate that social support was significantly associated with work value. Both social support and work value were significantly associated with job search behavior. The results of bias-corrected percentile bootstrap analysis revealed significant indirect effects of work value on the relationship between social support and job search behavior (Table 3).

**Table 2:** Summary table of mediation effect analysis.

	Model 1: Work value		Model 2: Job search behavior	
	β	*t*	β	*t*
Social support	0.21	3.56***	0.24	4.15***
Work value			0.12	2.04*
*R^2^*	0.06		0.07	
*F*	10.78***		17.21***	

*p < 0.05; ***p < 0.001.

**Table 3:** Path analysis results.

	Effect size	*SE*	*p*	95% CI
Total effect	0.65	0.02	<0.001	0.60, 0.71
Direct effect	0.48	0.02	<0.001	0.41, 0.55
Indirect effect	0.17	0.01	<0.001	0.15, 0.20

## Discussion

This study shows that job search behaviors are significantly influenced by both social support and work values. This study examined the impact of social support on job work-related behaviors based on the social exchange hypothesis. The outcomes are in line with the past empirical findings of Shirey (2004), Yu et al. (2014), Lee et al. (2017), and Yuh and Choi (2017). The investigation of the connection between social support and job search behavior during the COVID-19 epidemic is another important addition to the study. It is advised that job seekers with social support report less worry about their job search. It means that the social system has to be strengthened when looking for work since social interactions aid with career counseling and give useful information about possible career paths (Oludayo and Omonijo, 2020). These results concur with those of Kanfer et al. (2001), Van Hooft and Noordzij (2009), and Heydari et al. (2020). People who are active on social networks are less susceptible to the mental and emotional strain that unemployment causes (Heydari et al., 2020). Similar to this, receiving social support motivates someone to keep searching for work (Van Hooft et al., 2021). Additionally, having social connections when searching for a job helps to spread the pain and lessen the negative effects that come with it (Kanfer et al., 2001).

People look searching jobs that fit their unique combination of work values. Several researches have looked at the connection between students’ professional choices and their work values. For instance, Carruthers (1968) looked at how a sample of British students’ job search behavior and work values related. According to Cassar (2008), university teachers have an impact on students’ work values. However, Busacca et al. (2010) and Van Ness et al. (2010) discovered that the work value scores between practicing workers and trainees or students were considerably different. As a result, job experience also influences work values to some extent.

Third, the discovery that social support might influence job search behavior *via* work values broadens our comprehension of the process by which social support affects job search behavior. In line with earlier research, social support was able to significantly improve work values and job search behavior. It is important to note that this study discovered that social support has a stronger influence on job search behavior after work values. In other words, social support may change the value of a student’s effort, allowing them to benefit from social support elements like other people’s advice; it can also operate as a trigger to action, guiding students from the job search stage to the action phase of engaging in job search behaviors in the future.

## Conclusion

Despite the above limitations, the present study explored the mechanisms underlying the impact of social support on job search behavior, and revealed that work values mediate the relationship between social support and job search behavior It is suggested that providing support adequate support to college students’ for helping them procure employment will promote their positive employment. The school should provide the necessary support to the students in the form of employment training and guidance. In addition, the development of positive work values also plays an important and positive role in the employment of college students, so pertinent measures also need to be taken in this area.

### Practical implications

These findings provide useful advice for a range of stakeholders. First, it is advised to employ various group treatments to enhance social support for a job seeker since social support lowers job search anxiety. Regular family contacts aid job seekers in choosing the proper professional route with the necessary effort (Saltzman et al., 2020). For the engagement of final-year students, alumni, professionals in related fields, and volunteers, colleges and universities may construct social forums. It would make it easier for information, support, and therapy to flow. Second, the study’s findings suggest that work values influence job search behavior. This information is crucial for managers to understand what motivates staff members to stay with their jobs and could influence changes to management strategies that would improve working conditions and foster a supportive environment.

### Limitations

The current study offers a perspective on how social support influences job search behavior. However, there are still certain limitations that need to be overcome in future research. In order to have an in-depth understanding of the relationship between social support and job search behavior, extensive research is required. There may be other associations between social support and job seeking behavior which may exist. Furthermore, the convergent and discriminant validity of the scales should also be examined. In addition, this study employed a cross-sectional strategy; future research should use a longitudinal or experimental technique to corroborate the causal relationships.

## Data availability statement

The original contributions presented in the study are included in the article/supplementary material, further inquiries can be directed to the corresponding author.

## Author contributions

FW designed the study, performed the research, analyzed the data, and wrote the initial draft of the manuscript. J-GQ contributed to refining the ideas, carrying out additional analyses, and finalizing the manuscript. All authors contributed to the article and approved the submitted version.

## Conflict of interest

The authors declare that the research was conducted in the absence of any commercial or financial relationships that could be construed as a potential conflict of interest.

## Publisher’s note

All claims expressed in this article are solely those of the authors and do not necessarily represent those of their affiliated organizations, or those of the publisher, the editors and the reviewers. Any product that may be evaluated in this article, or claim that may be made by its manufacturer, is not guaranteed or endorsed by the publisher.
